# Retrospective study on the effect of adipose stem cell transplantation on jaw bone regeneration

**DOI:** 10.1186/s40729-024-00523-4

**Published:** 2024-02-05

**Authors:** Yasuhiro Kizu, Ryota Ishii, Naoyuki Matsumoto, Ichiro Saito

**Affiliations:** 1 Oral & Maxillofacial Care Clinic Yokohama, Kizu Dental Clinic, MM Grand Central Tower Bldg. 2F, 4-6-2, Minatomirai, Nishi-Ku, Yokohama, Kanagawa 220-0012 Japan; 2 Department of Oral Implantology, Tokyo Dental College, Tokyo, Japan; 3 Department of Oral Oncology Surgery, Tokyo Dental College, Tokyo, Japan; 4 Department of Pathology, Tsurumi University School of Dental Medicine, Yokohama, Kanagawa Japan

**Keywords:** Adipose stem cell transplantation, Jaw bone atrophy, Alveolar ridge augmentation, Bone regeneration, Dental implant therapy

## Abstract

**Purpose:**

In patients with jaw bone atrophy, dental implant therapy requires bone augmentation on the alveolar ridge. Common methods are autologous bone transplantation or bone substitutes. The latter technique is less surgically invasive because it does not require bone harvesting; however, blood supply from the surrounding tissues and local differentiation of osteoblasts are not guaranteed, so adequate bone regeneration for dental implant therapy is often not achieved. Therefore, at our hospital we introduced a bone regenerative medicine technique that uses adipose stem cells (ASCs) from adipose tissue. The new approach is less surgically invasive and appears to have a better effect on bone regeneration. The current retrospective study aimed to demonstrate the efficacy of ASC transplantation in patients who underwent alveolar ridge bone augmentation at our hospital.

**Methods:**

We compared medical records, postoperative radiographic findings, and histological results from patients treated between January 2018 and March 2022 by augmentation of the jaw bone with bone substitutes (carbonate apatite) mixed with ASCs (ASCs+ group) and those treated with bone substitutes (carbonate apatite) alone (ASCs− group).

**Results:**

After 6 months, the survival rate of augmented bone and the gray scale value in dental cone beam computed tomography (a bone density index) were significantly higher in the ASCs+ group than in the ASCs− group. Histological analysis at 6 months showed more adequate bone tissue regeneration in the ASCs+ group.

**Conclusions:**

The findings suggest the effectiveness of using ASCs in bone augmentation on the alveolar ridge in patients with jaw bone atrophy.

## Background

In dental implant therapy, autologous bone transplantation or tissue regeneration with bone substitutes is traditionally performed for jaw bones with severe atrophy or to treat periodontal disease with bone resorption. Of these two techniques, use of bone substitutes is preferred because harvesting autologous bone from sites such as the ilium is highly invasive. However, tissue regeneration is limited when using bone substitutes because they do not contain osteoblasts, blood vessels, and various other cells. Hence, research has evaluated the use of bone substitutes mixed with bone marrow-derived stem cells [1]. However, bone marrow contains very few stem cells [2], so interest has grown in adipose tissue as a source of adipose stem cells (ASCs) because it is rich in cell components and may produce good results when used with bone substitutes. Based on the above background, clinical research has evaluated the use of bone marrow-derived cells and cultured adipose tissue-derived cells in periodontal regeneration models and the regeneration of various organs and tissues [3, 4].

Some studies found that stem cells harvested from adipose tissue can be induced to differentiate into various mesodermal cells (e.g., bone, cartilage, muscle) in vitro [5, 6], and others reported that adipose tissue-derived stem cells, which are easily harvested, are effective for bone regeneration [7–9]. Non-cultured ASCs harvested by enzymatic treatment of human adipose tissue were shown to be rich in osteoblasts and neovessels [10, 11], so human cranial reconstruction was performed with such cells, and early skull restoration was reported [12]. Furthermore, ASCs and autologous fibrin glue (AFG) were used in reimplantation of cryopreserved skull fragments in patients with a wide calvarial defect after head injury, and complete continuity with the skull was shown 3 months after reconstruction [12]. In the field of jaw bone regeneration, a Dutch research group presented a summary of a protocol for regenerative therapy with ASCs and compared bone substitutes alone and bone substitutes mixed with ASCs for sinus floor bone augmentation in the bilateral maxillary molar regions before dental implant therapy [13]. The group performed bone biopsy during dental implant placement and found that bone substitutes mixed with ASCs achieved significantly better bone augmentation at the transplant site than the control did; no adverse events were reported [13, 14].

At our hospital, in 2019 we started using bone substitutes mixed with ASCs for bone augmentation on the alveolar ridge. To our knowledge, no report has described bone augmentation on the alveolar ridge in regions other than the maxillary molar region. Therefore, we performed a retrospective evaluation of patients treated with bone substitutes mixed with ASCs and those treated with conventional bone substitutes alone to demonstrate the efficacy of the former approach.

## Methods

### Participants

**Fig. 1:**
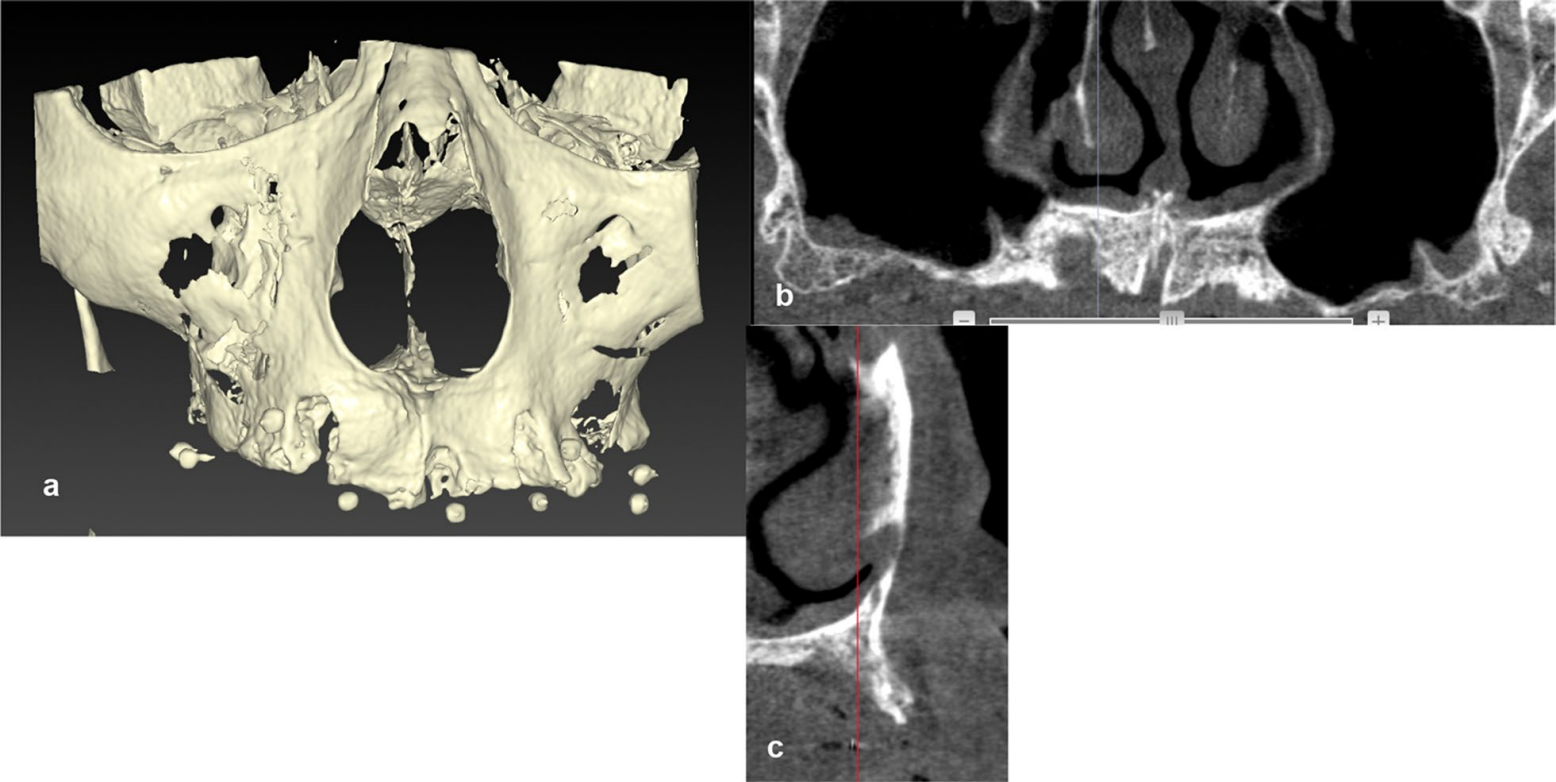
Representative findings in a patient with a jaw bone defect with bone substitutes (carbonate apatite) mixed with adipose stem cells. **a** Computed tomography image of a patient with extensive lateral bone defects in the jaw as representative findings of patients in the group treated with bone substitutes (carbonate apatite) mixed with adipose stem cells. **b** Extensive severe maxillary atrophies and defects. **c** Lateral bone defects with horizontal and vertical bone atrophies in anterior teeth

We analyzed medical records, postoperative radiographic findings, and histological results of patients with jaw bone atrophy who underwent alveolar ridge augmentation in the maxilla or mandible between January 2018 and March 2022 at the Oral & Maxillo-Facial Care Clinic, Yokohama, Japan, in preparation for receiving dental implant therapy. A total of 30 patients agreed to participate in this study and underwent or did not undergo ASC grafting for lateral bone defects. Patients were treated by bone augmentation with bone substitutes alone (carbonate apatite, CO_3_Ap; Cytrans®Granules) [15] (ASCs− group, *n* = 20) or with bone substitutes plus ASCs (ASCs+ group, *n* = 10). In both groups, patients with lateral jaw bone defects with fewer bone walls underwent horizontal and vertical lateral bone augmentation. In all cases, guided bone regeneration was performed in patients with similar bone defects of similar morphology. Patients with relatively extensive bone defects who required autologous transplantation of non-oral bone but agreed to treatment with the new approach to avoid autologous bone transplantation underwent bone augmentation with bone substitutes with ASC transplantation (Fig. 1). Other patients underwent conventional bone augmentation with bone substitutes without ASC transplantation. Patients excluded from alveolar ridge augmentation were those with systemic diseases that affect bone regeneration, such as diabetes, and those who were heavy smokers.

Exclusion criteria for this retrospective evaluation included being considered ineligible for participation because of the medical history, for example, and deciding not to participate after the study content was posted in the hospital.

This retrospective study was approved by the ethical review board of Tokyo Dental College on June 24, 2022 (medical ethical review number: 1118). Participants provided written informed consent for their data to be included in the study.

### Bone augmentation procedure

Patients in the ASCs+ group underwent bone augmentation with CO_3_Ap mixed with ASCs and filled with AFG; this treatment is certified as a type 2 regenerative medicine to be provided under the Act on Securing Safety of Regenerative Medicines in Japan (certification no. PB3170030). Patients in the ASCs− group underwent bone augmentation on the alveolar ridge with CO3Ap alone and filled with AFG.

To obtain ASCs, liposuction was performed by a plastic surgeon. First, under general anesthesia an adequate amount of tumescent solution (anesthetic solution: lactated Ringer’s solution 500 mL + 1% lidocaine 20 mL + 1 mg epinephrine) was infused into the subcutaneous adipose tissue at the adipose tissue harvest site (abdomen or thigh). Approximately 30 min after infusion of the solution, a suspension containing adipose tissues was manually aspirated with a dedicated syringe and cannula.

**Fig. 2:**
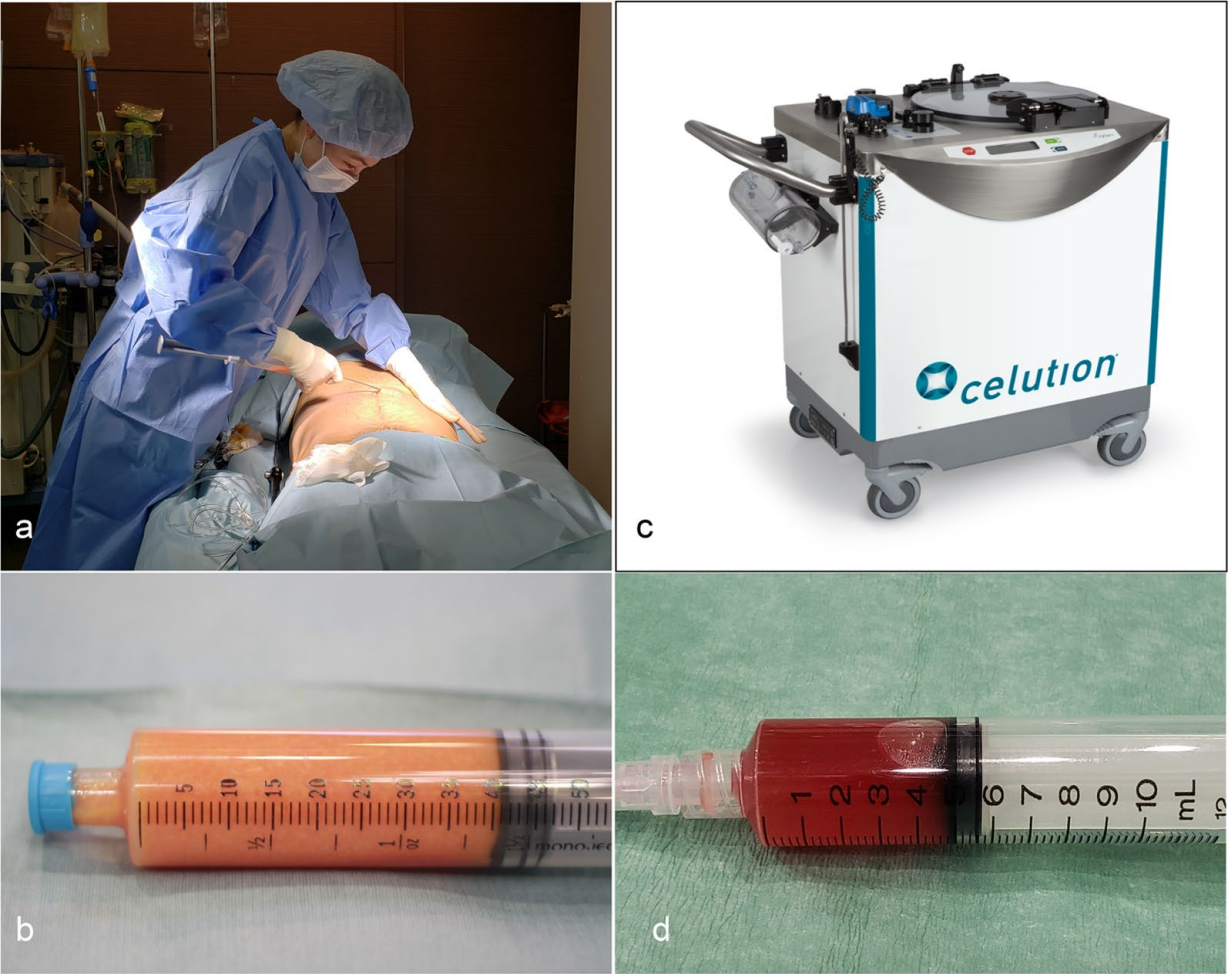
Extraction of adipose stem cells from harvested adipose tissue. **a** A plastic surgeon harvesting adipose tissue (100 to 200 g) by liposuction. **b** Harvested adipose tissue. **c** Celution 800/CRS device (Cytori Therapeutics, Inc., San Diego, CA, USA), which was used to extract adipose stem cells from harvested adipose tissue. **d** Cell suspension containing extracted ASCs (about 5 mL)

After removal of the adipose tissue (100 to 250 mL; mean, 171 mL), the tissue was washed with lactated Ringer’s solution, and ASCs were separated from adipose tissue with a Celution 800/CRS device (Cytori Therapeutics, Inc., San Diego, CA, USA). After separation, the cell suspension containing ASCs (about 5 mL) was extracted with a centrifuge (Fig. 2). A small amount of the cell suspension was used for assessment of cell viability and measurement of the number of living cells with a Nucleocounter NC-100 (ChemoMetec A/S, Allerod, Denmark). The optimal characteristics for cell transplantation therapy were set as a cell viability of 70% or higher and a minimum concentration of living cells of 1 × 10^6^/5 mL [9].

**Fig. 3:**
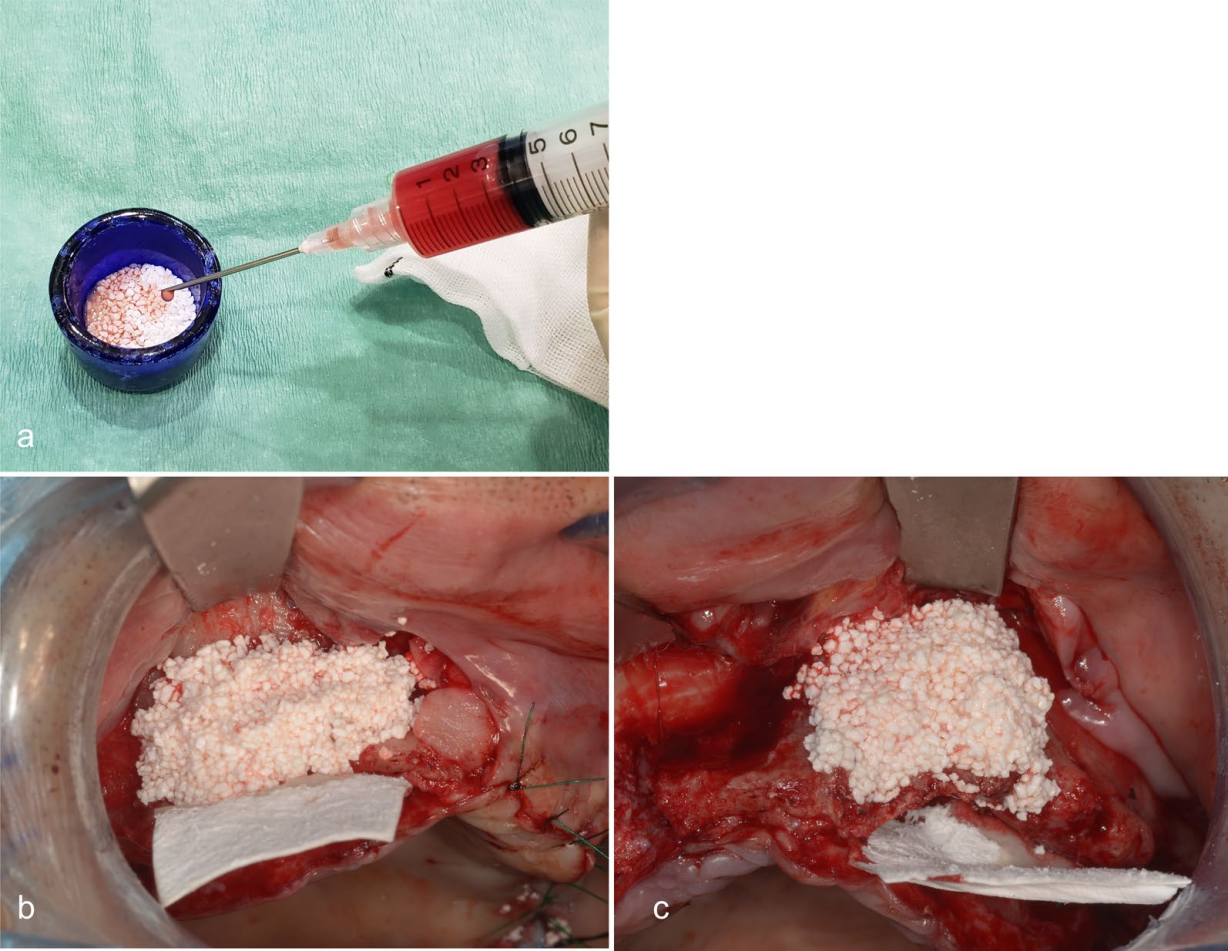
Bone augmentation on the alveolar ridge with grafts mixed with adipose stem cells and bone substitutes (carbonate apatite). Extracted adipose stem cells (ASCs) were mixed with bone substitutes (carbonate apatite) (**a**) and bone augmentation was performed at sites 13 to 11 and 21 to 23 with grafts mixed with ASCs (**b**, **c**)

Jaw bone augmentation in the maxillary and/or mandibular alveolar ridges was performed by an oral surgeon. Bone augmentation was performed by transplanting a graft with bone substitutes mixed with ASCs (ASCs+ group; Fig. 3) or not (ASCs− group) and further filled with AFG.

### Evaluation of bone regeneration

**Fig. 4:**
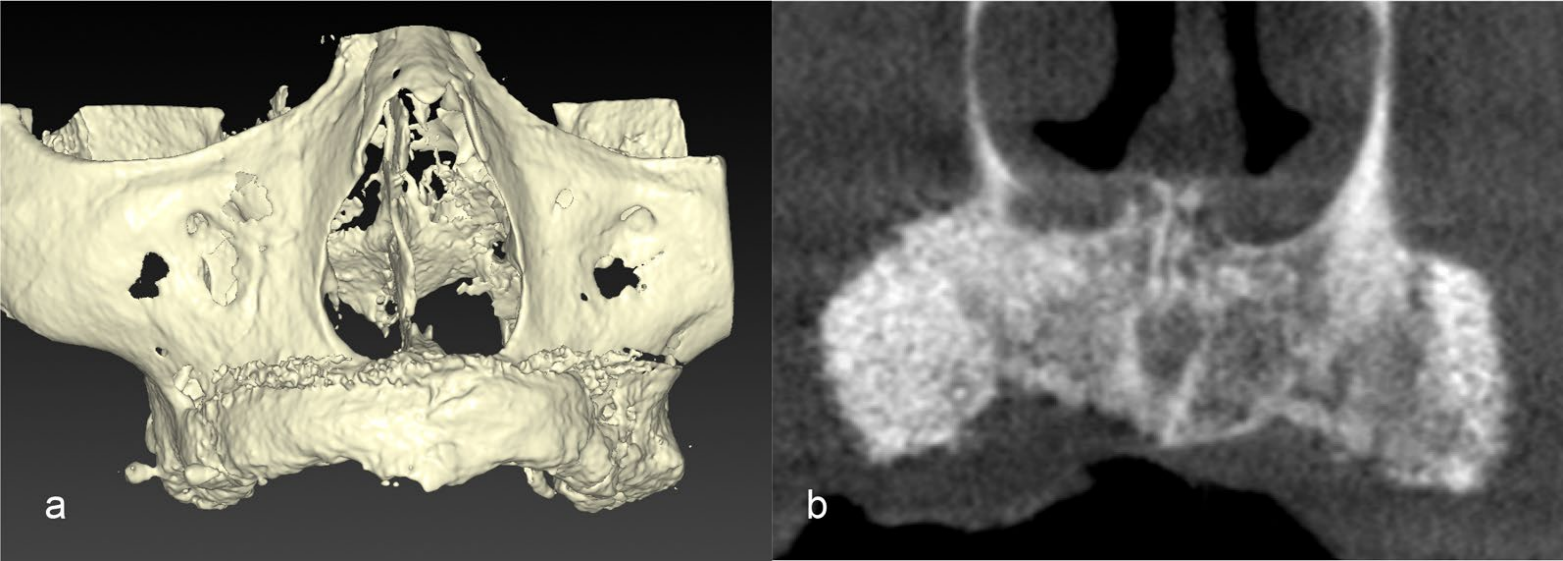
Cone beam computed tomography findings immediately after bone augmentation with adipose stem cells and bone substitutes (carbonate apatite). Three-dimensional image (**a**) and frontal plane image (**b**) 6 months after bone augmentation showing high gray scale values of bone augmentation

**Fig. 5:**
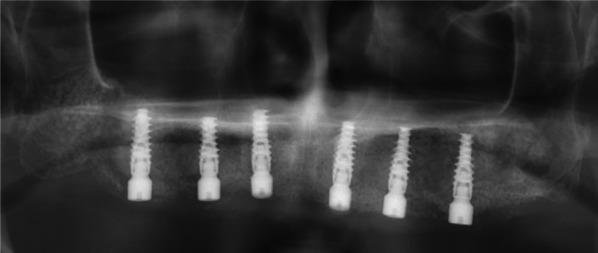
Panoramic X-ray image after implant placement. Six implants were placed in augmented bone

**Fig. 6:**
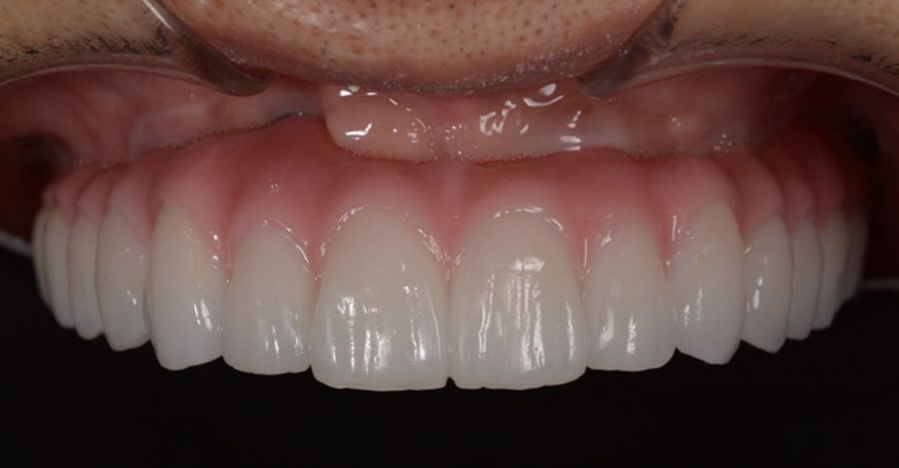
Screw-retained prosthesis. The prosthesis was installed approximately 6 months after placement of implants into the augmented bone

In both the ASCs+ and ASCs− groups, a dental cone beam computed tomography (CT) scan was performed approximately 6 months after bone augmentation to confirm bone regeneration (Fig. 4). Then, an oral surgeon performed dental implant placement (Fig. 5). After approximately 6 months, a screw-retained prosthesis was installed (Fig. 6).

To evaluate the change in the amount of regenerated bone around the site of the dental implant, the height of the augmented bone was measured during the immediate postoperative period after bone augmentation and at 6 months postoperatively from CT images. The height of the augmented bone was measured at the site about 1 mm horizontally from the implant site, and the mean height was calculated. The bone survival rate was calculated from the height of the augmented bone during the immediate postoperative period after bone augmentation or at 6 months postoperatively.

To measure the bone density around the dental implant site, gray scale (GS) values were measured in the cone beam CT scan during the immediate postoperative period and at 6 months postoperatively. Measurements were made at 3 points on the same horizontal plane, 1 mm, 3 mm, and 5 mm from the implant site, and vertically from the existing bone on the alveolar ridge, and the means were calculated.

The cone beam CT system was ProMax 3D Mid with Romexis Ver: 4.6.2 software (Planmeca, Finland). The measured GS values were the values displayed as the mean of the Hounsfield Unit reference values in the 3 × 3 pixel range on the screen. The parameters of the CT image GS were as follows: bit number, 15; window, 2500; level, 2500.

Metal artifacts around the implant body are problematic when measuring GS values. Therefore, GS values were measured at a distance of 1 mm horizontally from the implant body after artifacts were removed by image processing with the Romexis Ver. 4.6.2 software of the cone beam CT system.

On the other hand, although GS values are a valid way to evaluate images because they measure the intensity of displayed pixels, it is difficult to use them to accurately evaluate the quality of the jawbone. Therefore, the quality of the regenerated bone was evaluated histologically.

**Fig. 7:**
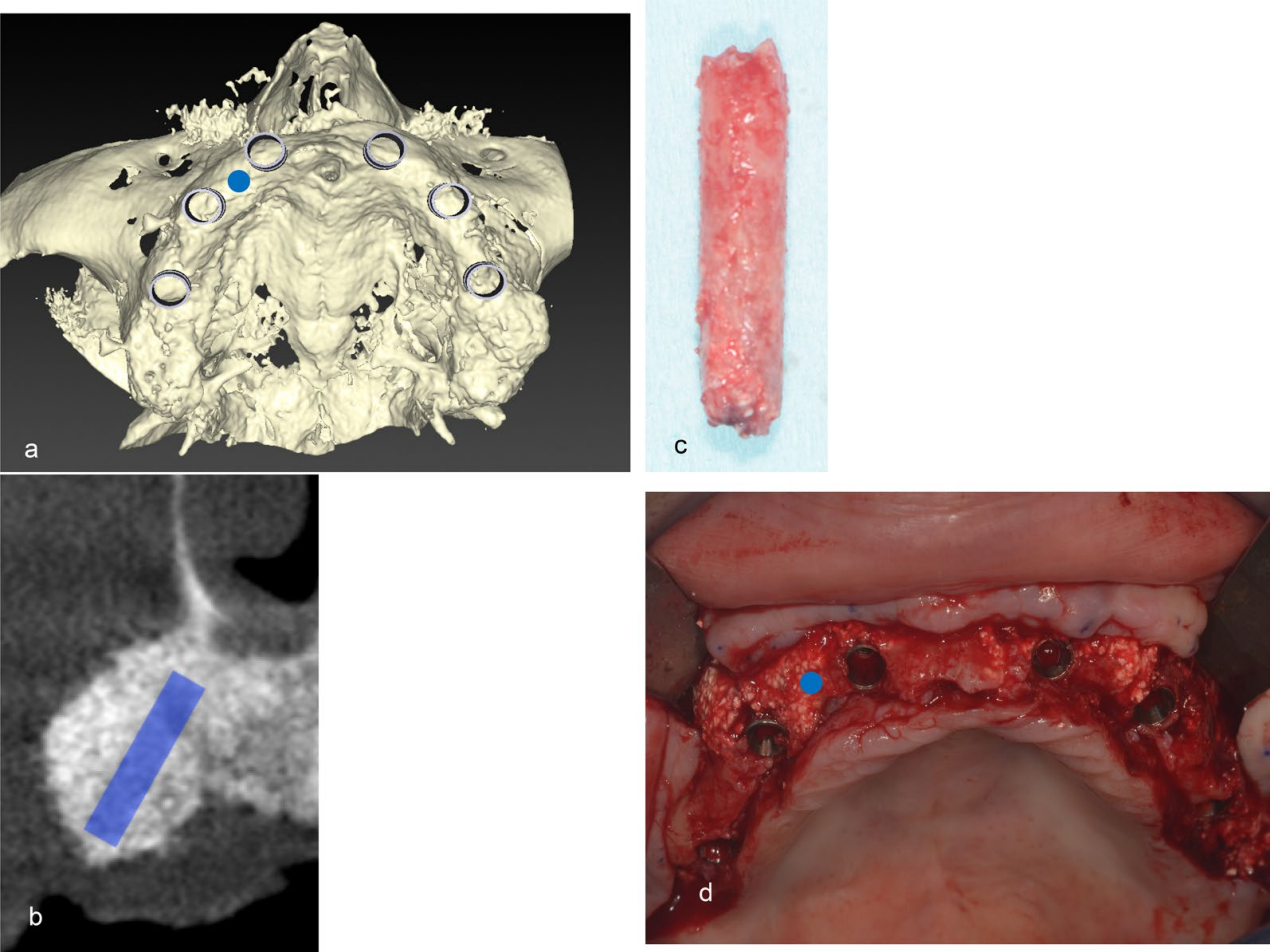
Computed tomography images of sites where augmented bone was harvested. During implant placement 6 months after bone augmentation, 3-dimensional (**a**) and sagittal plane (**b**) computed tomography images of bone harvest sites were used to assist harvesting of augmented bone with a trephine bur (**c**) at the site related to tooth number 13. The bone harvest site is the blue circle in **a** and **c** and blue bar in **b**. Harvested augmented bone (**d**)

Six months after bone augmentation, a CT image of the maxilla was obtained (Fig. 7a, b) to assist in harvesting a trephine bur (Fig. 7c, d) as an augmentation specimen for histological analysis as part of the treatment. The removed bone tissues were fixed with 4% paraformaldehyde-phosphate buffered saline (PBS, Nacalai Tesque, Inc., Kyoto, Japan) for 1 week and then decalcified with 0.5 M ethylenediaminetetraacetic acid (Dojindo Laboratories, Kumamoto, Japan)-PBS (pH 7.2) for 4 weeks. Subsequently, the decalcified bone tissues were cut into two pieces along a longitudinal axis and embedded in a paraffin block. Then, the paraffin blocks were cut into 4-μm-thick slices, mounted on a slide glass, and stained with hematoxylin and eosin (HE). Photographs of the HE-stained specimens of augmented bone areas were captured with a fluorescence microscope (BZ-9000, Keyence Corporation, Osaka, Japan) under normal and fluorescent (excitation, 470/40 nm; emission, 525/50 nm) light. Under fluorescent light, bone tissue areas were selectively brighter than the background [16]. The total area (T.Ar) and bone area (B.Ar) [17] were measured with ImageJ, and histological bone density was calculated as B.Ar/T.Ar (%) [18].

### Statistical analysis

Normality and homoscedasticity were analyzed before statistical analyses were performed. To compare two groups or conditions, we used the Mann–Whitney *U* test, and to compare three groups or conditions, we used the Kruskal–Wallis *H* test and confirmed the results by the Mann–Whitney *U* test with Bonferroni correction. All statistical analyses were performed with ystat2013 (an Excel® statistical program file, which was programmed by Dr. Yamazaki, Ohu University, Fukushima, Japan). Results are presented as means. *P* values of less than 0.05 were considered statistically significant.

## Results

The study included 10 patients (4 men, 6 women) in the ASCs+ group (mean age, 60.4 years; range, 46–71 years) and 20 patients (8 men, 12 women) in the ASCs− group (mean age, 59.5 years; range, 41–77 years). In the ASCs+ group, 8 patients underwent maxillary augmentation and 4, mandibular augmentation, and in the ASCs− group, 12 patients underwent maxillary augmentation and 8, mandibular augmentation. Information on patients in the ASCs+ group and ASCs− group is shown in Table 1. No systemic diseases occurred in any patients. Blood tests performed 1 month postoperatively showed no abnormal values in any of the patients.

**Table 1:** Information on patients who underwent jaw bone augmentation with bone substitutes (carbonate apatite) with or without adipose stem cells

Patient number	Sex	Age (years)	ASCs^a^	Site of tissue collection	Aspirated amount of adipose tissue (mL)	Dental implant sites^b^
1	Male	52	+	Abdomen	190	12,14,22,24
2	Female	46	+	Thigh	270	12,14,22,24,
3	Female	69	+	Abdomen	150	45,46,47
4	Female	71	+	Thigh	150	12,13,22,23
5	Male	65	+	Abdomen	100	33,35,37,43,45,47
6	Female	52	+	Thigh	205	11,13,14,23,24,
7	Female	65	+	Thigh	135	12,21,23,24,47
8	Male	65	+	Thigh	135	12,14,22,24,
9	Female	68	+	Thigh	205	12,14,22,24
10	Male	51	+	Thigh	170	16,17,31,34,36,37,42,47
11	Female	62	−	–	–	22
12	Male	68	−	–	–	12,22
13	Female	41	−	–	–	11,22
14	Female	64	−	–	–	12,14,22,24
15	Female	41	−	–	–	21
16	Male	54	−	–	–	11,21
17	Female	43	−	–	–	11,22
18	Female	41	−	–	–	11
19	Female	71	−	–	–	13,15,21,23,25
20	Male	60	−	–	–	15
21	Female	56	−	–	–	14
22	Male	77	−	–	–	16,17
23	Female	57	−	–	–	36
24	Male	44	−	–	–	36
25	Female	67	−	–	–	45,47
26	Male	47	−	–	–	36
27	Female	56	−	–	–	45,47
28	Male	60	−	–	–	36,37
29	Male	57	−	–	–	47
30	Female	60	−	–	–	46,47

^a^ASCs, adipose stem cells; ASCs+, group with adipose stem cells; ASC−, group without adipose stem cells

^b^Dental implant sites are named according to the Fédération Dentaire Internationale (FDI) system

### Viability of cells extracted with the Celution 800/CRS device and number of living cells

The cell viability was 89.4% to 95.2% (mean, 92.7%) and the number of living cells was 6.62 × 10^6^ to 113.0 × 10^6^ cells (mean 40.5 × 10^6^)/5 mL, so the harvest efficiency was high enough to perform cell transplantation therapy.

### Results of bone augmentation

In the ASCs+ group, the vertical height of the regenerated bone was measured 31 times in 8 patients who underwent maxillary augmentation and 16 times in 4 patients who underwent mandibular augmentation. In the ASCs− group, it was measured 24 times at 6 months postoperatively in 12 patients who underwent maxillary augmentation and 12 times at 6 months postoperatively in 8 patients who underwent mandibular augmentation.

### Survival rate of augmented bone (height)

**Fig. 8:**
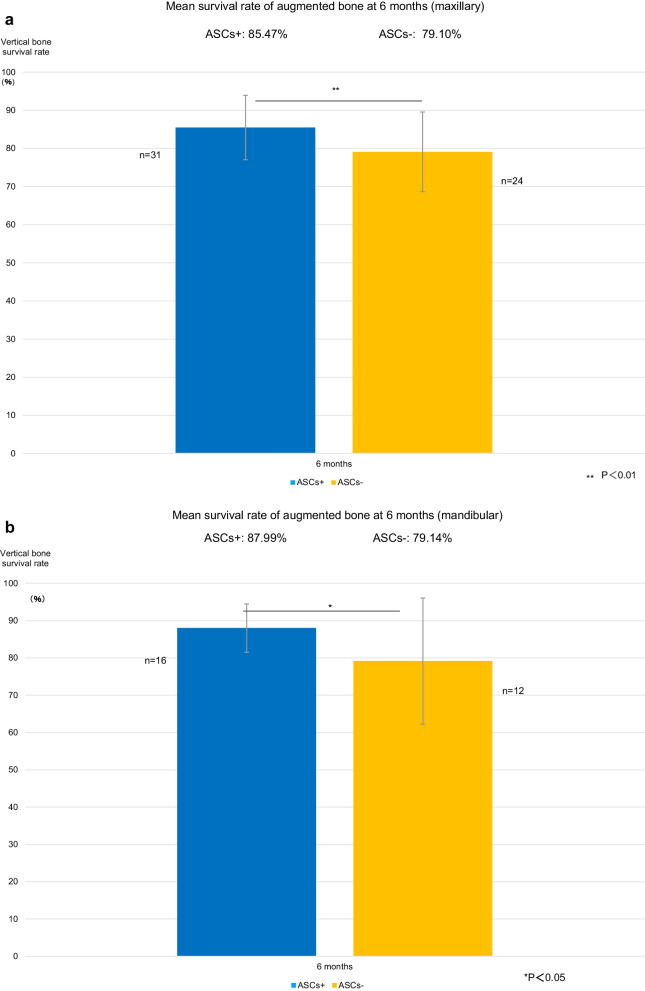
Vertical bone survival rates. **a** Vertical bone survival rate after maxillary augmentation. At 6 months postoperatively, the bone survival rate was significantly higher in the group that received bone substitutes mixed with adipose stem cells (ASCs+ group) than in the group that received bone substitutes alone (ASCs− group) (**P < 0.01). **b** Vertical bone survival rate after mandibular augmentation. At 6 months postoperatively, the bone survival rate was significantly higher in the group that received bone substitutes mixed with adipose stem cells (ASCs+ group) than in the group that received bone substitutes alone (ASCs− group) (**P < 0.01). ASCs+, bone substitutes mixed with adipose stem cells; ASCs−, bone substitutes without adipose stem cells

The mean height of the bone augmentation was 10.33 mm in the ASCs+ group and 9.44 mm in the ASCs− group immediately after maxillary augmentation and 8.78 mm and 7.47 mm, respectively, at 6 months. The bone survival rate was 85.5% in the ASCs+ group and 79.1% in the ASCs− group and was significantly higher in the ASCs+ group than in the ASCs− group (*P* < 0.01) (Fig. 8a).

The mean height of bone augmentation was 7.16 mm in the ASCs+ group and 7.82 mm in the ASCs− group immediately after mandibular augmentation and 6.28 mm and 6.14 mm, respectively, at 6 months. The bone survival rate was 88.0% in the ASCs+ group and 79.1% in the ASCs− group at 6 months and was significantly higher in the ASCs+ group than in the ASCs− group (*P* < 0.05) (Fig. 8b).

### GS values in bone augmentation

In the ASCs+ group, the number of measurements of GS values was 31 (8 patients) after maxillary augmentation and 16 (4 patients) after mandibular augmentation. In the ASCs− group, the number was 24 (12 patients) after maxillary augmentation and 12 (8 patients) after mandibular augmentation.

Maxillary GS values at the site 1 mm vertically from the existing bone were 816.3 for the ASCs+ group and 823.3 for the ASCs− group during the immediate postoperative period after bone augmentation and 1487.7 and 1206.2, respectively, at 6 months; those at 3 mm were 821.2 and 821.8, respectively, during the immediate postoperative period and 1518.4 and 1270.3, respectively, at 6 months; and those at 5 mm were 820.2 and 820.4, respectively, during the immediate postoperative period and 1565.2 and 1290.8, respectively, at 6 months.

The corresponding mandibular GS values were as follows: at the site 1 mm vertically from the existing bone, 817.9 for the ASCs+ group and 809.4 for the ASCs− group during the immediate postoperative period and 1441.7 and 1156.3, respectively, at 6 months; at 3 mm, 817.8 and 797.7, respectively, during the immediate postoperative period and 1454.8 and 1226.2, respectively, at 6 months; and at 5 mm, 817.8 and 801.9, respectively, during the immediate postoperative period and 1465.8 and 1222.8, respectively, at 6 months.

**Fig. 9:**
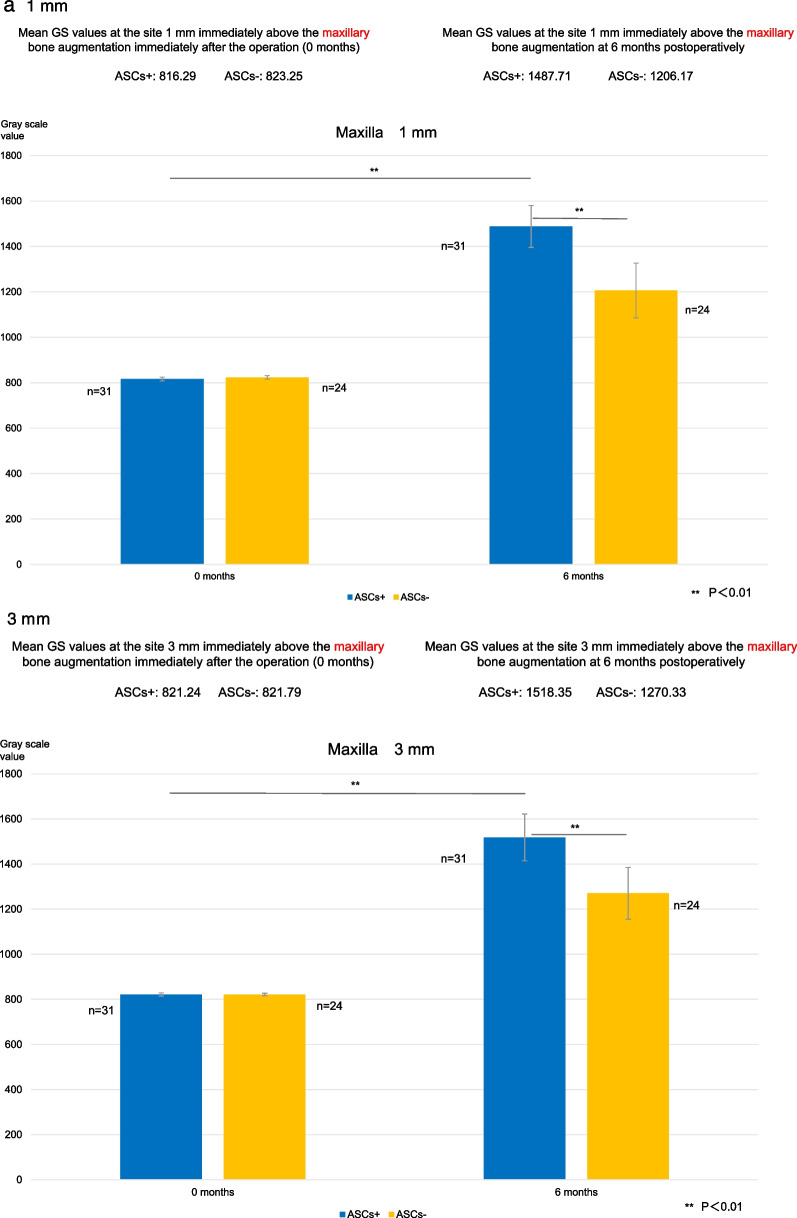

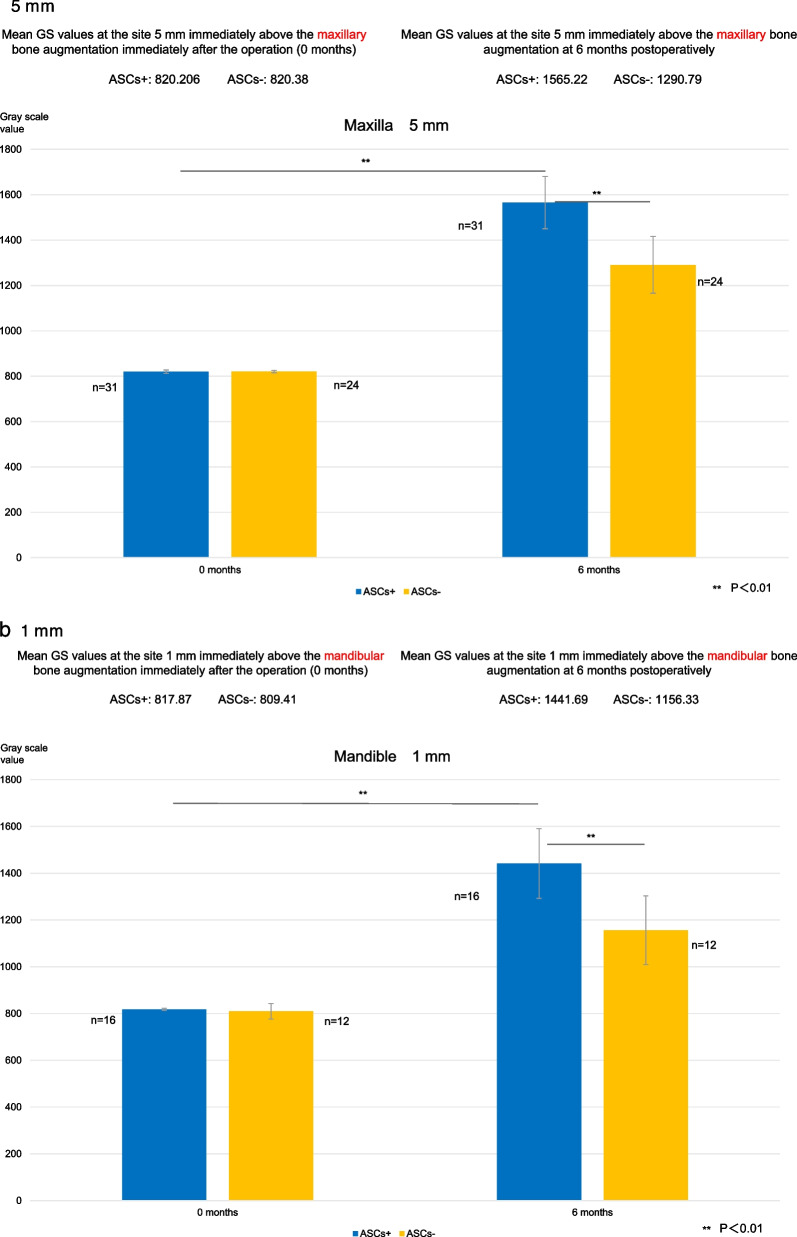

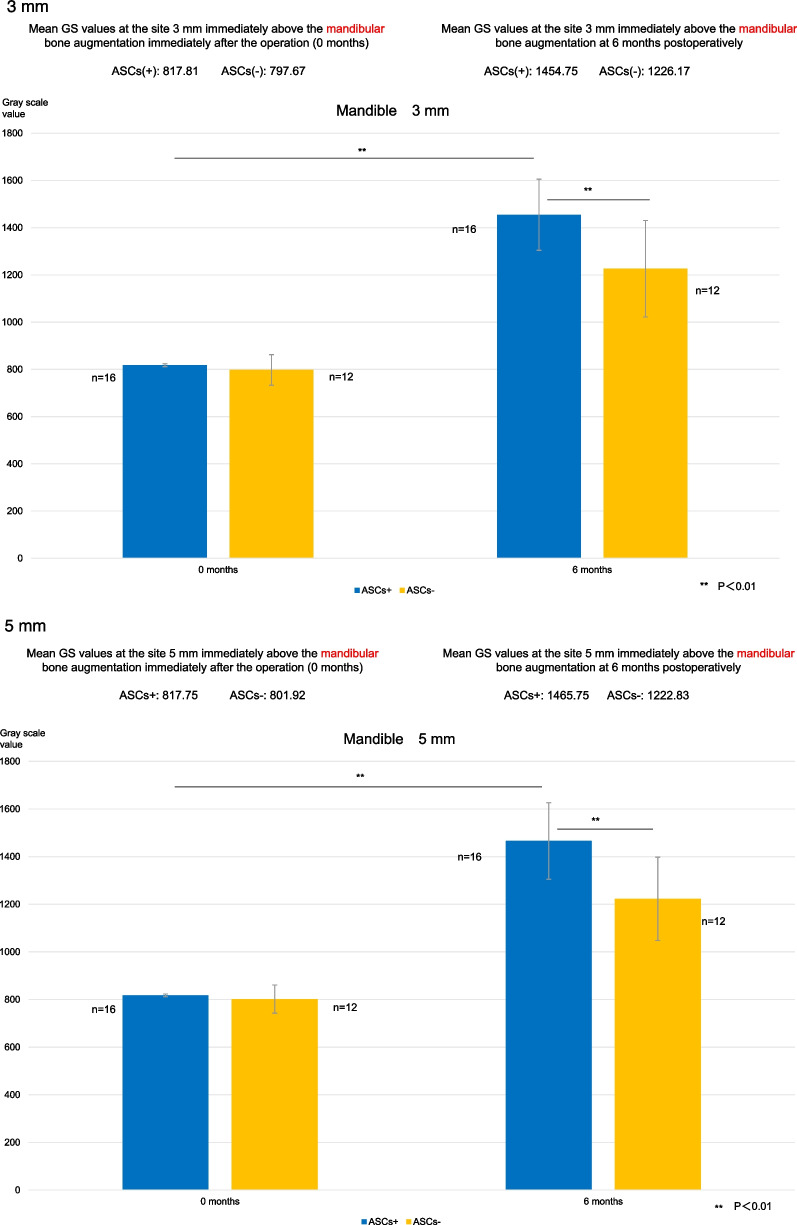
Gray scale values. **a**. Gray scale values at the sites 1 mm, 3 mm, and 5 mm immediately above the maxillary bone augmentation. At 6 months postoperatively, gray scale values at all measurement sites were significantly higher in the group that received bone substitutes mixed with adipose stem cells (ASCs+ group) than in the ASCs+ group immediately after the operation (0 months) and at all postoperative measurement time points in the group that received bone substitutes alone (ASCs− group) (**P < 0.01). **b** Gray scale values at the sites 1 mm, 3 mm, and 5 mm immediately above the mandibular bone augmentation. At 6 months postoperatively, gray scale values at all measurement sites were significantly higher in the group that received bone substitutes mixed with adipose stem cells (ASCs+ group) than in the ASCs+ group immediately after the operation (0 months) and at all postoperative measurement time points in the group that received bone substitutes alone (ASCs− group) (**P < 0.01). ASCs+, bone substitutes mixed with adipose stem cells; ASCs−, bone substitutes without adipose stem cells; GS, gray scale

Changes in GS values after maxillary augmentation are shown in Fig. 9a. The percentage increases in the GS value at 6 months were as follows: at the site 1 mm vertically from the existing bone, 182.2% in the ASCs+ group and 146.5% in the ASCs− group; at the site 3 mm vertically from the existing bone, 184.9% in the ASCs+ group and 154.6% in the ASCs− group; and at the site 5 mm from the existing bone, 190.9% in the ASCs+ group and 157.3% in the ASCs− group. All measurements of GS values at 6 months were significantly higher in the ASCs+ group than in the ASCs− group (all *P* < 0.01). In both groups, GS values were significantly higher at 6 months than during the immediate postoperative period (all *P* < 0.01).

Changes in GS values after mandibular augmentation are shown in Fig. 9b. The percentage increase in the GS value at 6 months was 176.3% in the ASCs+ group and 142.9% in the ASCs− group at the site 1 mm vertically from the existing bone; 177.9% in the ASCs+ group and 153.7% in the ASCs− group at the site 3 mm vertically from the existing bone; and 179.2% in the ASCs+ group and 152.5% in the ASCs− group at the site 5 mm from the existing bone. All measurements of GS values at 6 months were significantly higher in the ASCs+ group than in the ASCs− group (all *P* < 0.01). In both groups, GS values were significantly higher at 6 months than during the immediate postoperative period (all *P* < 0.01).

### Histological evaluation of augmented bone

#### *Representative histological images are shown for ASCs*+ *and ASCs− groups*

**Fig. 10:**
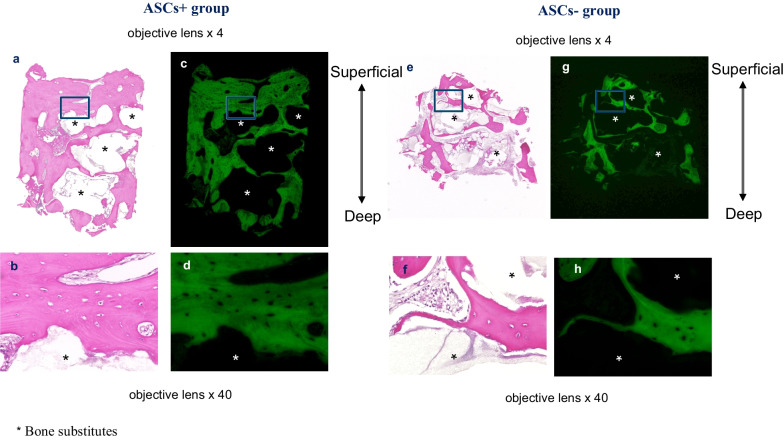
Bone tissue histopathology. Biopsy specimens of augmented bone tissue taken at 6 months postoperatively showed bone tissue formation around the bone replacement material in both the group that received bone substitutes mixed with adipose stem cells (ASCs+ group) (**a**–**d**) and in the group that received bone substitutes alone (ASC− group) (**e**–**h**). However, there was more bone formation around the bone replacement material (*) in the ASCs+ group than in the ASCs− group. ASCs+, bone substitutes mixed with adipose stem cells; ASCs−, bone substitutes without adipose stem cells

**Fig. 11:**
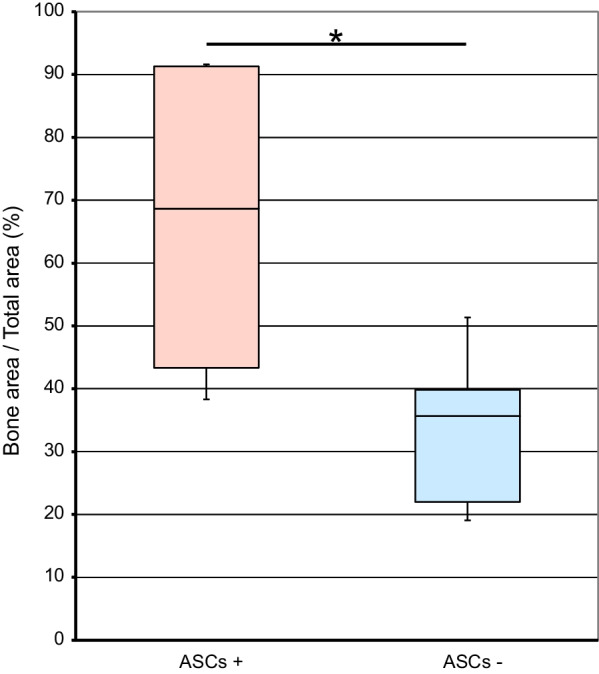
Histological bone density. Histological bone density was significantly higher in the ASCs+ group than in the ASCs− (66.0% ± 29.8 vs 33.7% ± 13.8, respectively). *P < 0.05. ASCs+, bone substitutes mixed with adipose stem cells; ASCs−, bone substitutes without adipose stem cells

Bone tissue specimens were taken 6 months after bone augmentation from bone substitutes mixed with adipose stem cells (ASCs+ group, a–d) and after bone augmentation with bone substitutes alone (ASCs− group, e–h). Hematoxylin and eosin-stained specimens were examined under normal light (a, b, e, f) and fluorescent light (c, d, g, h). Bone formation was observed around bone substitutes (*) in the ASCs+ (a–d) and ASC− (e–h) groups. However, there was more bone formation around the bone replacement material in the ASCs+ group than in the ASCs− group (Fig. 10). The histological bone density was significantly higher in the ASCs+ group than in the ASCs− group (66.0% ± 29.8 vs 33.7% ± 13.8, respectively; *P* < 0.05) (Fig. 11).

## Discussion

ASCs have no risk of transplant rejection and a low risk of tumorigenesis because they are autologous cells. Furthermore, because ASCs are extracted from autologous tissue, there is no risk of contamination, unlike with cultured cells. In addition, the relatively easy sampling process reduces the burden on the patient. ASCs have been used in many diseases, such as cerebral infarction, liver cirrhosis, and dementia, and for esthetic restoration. However, the liquid component produced by ASCs in individuals with obesity (BMI > 30) may promote cancer cell proliferation, so ASC transplantation is contraindicated in such individuals. It is also contraindicated in cancer patients because of its reported effect on tumorigenesis and tumor progression [19]. Thus, care should be taken not to perform this procedure in patients with risks or contraindications.

The present study showed significant bone regeneration associated with bone augmentation on the alveolar ridge in terms of the height of the augmented bone and bone density in the ASCs+ group compared with the ASCs− group. Usually, bone augmentation on the alveolar ridge with bone substitutes alone occurs only laterally, which has a negative effect on bone regeneration because poor blood flow in the cortical bone of the jaw makes it difficult for stem cells and osteoblasts to migrate along microvessels generated in the scaffold created by bone substitute. Therefore, addition of ASCs to bone substitutes may increase blood flow from capillaries of surrounding bone tissue and induce the MSCs necessary for bone tissue regeneration [20]. In addition, some studies found that ASCs differentiate into platelets, which support tissue repair and regeneration [21, 22]. These studies suggest that ASC transplantation may increase blood flow from surrounding capillaries, leading to migration of MSCs to bone regeneration sites and effective bone tissue regeneration associated with the function of platelets generated from ASCs [20–22].

In the present study, to anchor stem cells for bone augmentation, harvested ASCs were added to bone substitutes and mixed with AFG. Stefan et al. reported that mixing ASCs with AFG in re-transplantation of cryopreserved skull fragments in patients with a widespread calvarial defect allowed ASCs to be anchored in sites planned for regeneration [12]. Because of its biocompatibility and biodegradability, AFG may not inhibit stem cell growth, so it is effective for anchoring stem cells [23]. Furthermore, it is known to function as a biological mediator for transplanted cells [24]. Thus, besides anchoring ASCs, mixing bone substitutes with ASCs and AFG potentially induces differentiation of ASCs and peri-augmentation of MSCs into osteoblasts because of stimulation by various growth factors and cytokines in AFG. Research has also shown that, during remodeling, fibroblast growth factor-2 strongly stimulates MSCs [25]. Thus, the presence of elements for tissue regeneration, such as stem cells in surrounding bone induced by ASC transplantation, ASCs, and AFG containing cytokines (growth factors), together with bone substitutes as a scaffold, would maintain the morphology and quality of bone tissue during bone regeneration and effectively support dental implant therapy. In particular, humoral components from tissue stem cells are reported to promote tissue regeneration [26] and cytokines such as osteoprotegerin [27, 28] and vascular endothelial growth factor [29, 30], humoral factors secreted from ASCs, have been reported in recent years to support bone formation. Such growth factors may act on existing osteoblasts to stimulate bone formation, but the detailed mechanisms require additional study.

This study has two main limitations. First, the study sample was small, so the findings need to be confirmed in a larger sample in the future. And second, in vitro studies are needed to investigate the mechanism of bone regeneration in ASCs.

Patients who undergo jaw osteotomy because of severe atrophy of the jaw bone or tumors currently have no other choice but to undergo alveolar ridge augmentation with autologous bone transplantation (e.g., from the ilium), which is a highly invasive procedure. Jaw bone augmentation with ASC transplantation and bone substitutes may achieve effective bone regeneration also in these patients, and further study is warranted.

## Conclusions

This retrospective study showed that bone augmentation on the alveolar ridge with bone substitutes and ASCs achieves better bone regeneration results than augmentation with bone substitutes alone. The addition of ASCs achieves higher density of augmented bone and less loss of generated bone over time compared with the traditional method. These results suggest that ASC transplantation is an effective approach for alveolar ridge augmentation and can thus improve dental implant therapy.

## Data Availability

The datasets used and/or analyzed during the current study are available from the corresponding author on reasonable request.

## Abbreviations

AFG: Autologous fibrin glue
ASC: Adipose stem cell
CT: Computed tomography
GS: Gray scale
